# Microbes at Surface-Air Interfaces: The Metabolic Harnessing of Relative Humidity, Surface Hygroscopicity, and Oligotrophy for Resilience

**DOI:** 10.3389/fmicb.2016.01563

**Published:** 2016-09-30

**Authors:** Wendy Stone, Otini Kroukamp, Darren R. Korber, Jennifer McKelvie, Gideon M. Wolfaardt

**Affiliations:** ^1^Department of Microbiology, University of Stellenbosch, Cape TownSouth Africa; ^2^Department of Chemistry and Biology, Ryerson University, Toronto, ONCanada; ^3^Department of Food and Bioproduct Sciences, University of Saskatchewan, Saskatoon, SKCanada; ^4^Environmental Geoscience, Nuclear Waste Management Organization, Toronto, ONCanada

**Keywords:** desiccation, metabolism, relative humidity, surface, carbon

## Abstract

The human environment is predominantly not aqueous, and microbes are ubiquitous at the surface-air interfaces with which we interact. Yet microbial studies at surface-air interfaces are largely survival-oriented, whilst microbial metabolism has overwhelmingly been investigated from the perspective of liquid saturation. This study explored microbial survival and metabolism under desiccation, particularly the influence of relative humidity (RH), surface hygroscopicity, and nutrient availability on the interchange between these two phenomena. The combination of a hygroscopic matrix (i.e., clay or 4,000 MW polyethylene glycol) and high RH resulted in persistent measurable microbial metabolism during desiccation. In contrast, no microbial metabolism was detected at (a) hygroscopic interfaces at low RH, and (b) less hygroscopic interfaces (i.e., sand and plastic/glass) at high or low RH. Cell survival was conversely inhibited at high RH and promoted at low RH, irrespective of surface hygroscopicity. Based on this demonstration of metabolic persistence and survival inhibition at high RH, it was proposed that biofilm metabolic rates might inversely influence whole-biofilm resilience, with ‘resilience’ defined in this study as a biofilm’s capacity to recover from desiccation. The concept of whole-biofilm resilience being promoted by oligotrophy was supported in desiccation-tolerant *Arthrobacter* spp. biofilms, but not in desiccation-sensitive *Pseudomonas aeruginosa* biofilms. The ability of microbes to interact with surfaces to harness water vapor during desiccation was demonstrated, and potentially to harness oligotrophy (the most ubiquitous natural condition facing microbes) for adaptation to desiccation.

## Introduction

Water is widely considered the determining element of the metabolic network that constitutes life on earth (Zolensky, 2005). In the microbial context, one of the earlier fundamental reviews on water activity in soil underscored this by calling single-cellular microbes aquatic creatures, regardless of their habitat (Stotzky and Pramer, 1972). Similarly, Brown articulated that, since prokaryotes predominantly obtain nutrients from solution, even a desiccated environment is essentially just a “concentrated solution with which the microbial cell must come directly to thermodynamic terms” in order to persist (Brown, 1976). These observations highlight a bias toward liquid systems that has pervaded microbiological research for decades. However, despite the methodological challenges of studying microbial activity under desiccation, the environments with which humans interact are primarily not aquatic. Also, microbes are renowned for their ubiquity in terrestrial niches and flexibility in response to diverse stressors, many of which are often simultaneously associated with desiccation (Chang and Halverson, 2003; Ramirez et al., 2004; Ritchie et al., 2006; Bauermeister et al., 2011; de Goffau et al., 2011). Thus, although it is true that microbial activity and survival at surface-air interfaces must be explored in relation to the essential element water, the study of their activity in these inherently slower desiccated conditions is not anomalous, but rather a truer exploration of how microbes persist and influence many industrial, agricultural, and medical settings relevant to humans. Examples of such “slow” surface-air microbiome consequences include the weathering of rock (Gorbushina and Broughton, 2009; de Goffau et al., 2011), structural architecture (Mottershead et al., 2003; Cheng et al., 2016) and artwork (Guglielminetti et al., 1994; Garg et al., 1995), hospital pathogen persistence (Kramer et al., 2006), risk-assessment models for the International Space Station (de Goffau, 2011), solid food contamination (Brown, 1976) and agricultural soil ecology (Stotzky and Pramer, 1972; Ramirez et al., 2004; Ritchie et al., 2006). Microbiology at surface-air interfaces also has potential positive impacts, including the turnover of greenhouse gasses by soil microbes (Lammel et al., 2015). This was harnessed via biomimicry, with the immobilization of methane-oxidizing bacteria on building materials acting as a carbon sink and removing methane from the air (Ganendra et al., 2014). Ecologically, microbial diversity has been demonstrated in dust storms (Katra et al., 2014) and microbes can travel great distances whilst maintaining metabolic viability (Meola et al., 2015).

Spore-formation, and the accompanying metabolic pause, is arguably the best-studied response of microbes to desiccation (de Goffau et al., 2011; Feofilova et al., 2012). The regulated molecular responses to various stressors (osmotic, heat, or cold) have been extensively elucidated, and include a cascade of sigma transcription factors, heat and cold shock proteins, and RNA chaperones (Biran and Ron, 2016). Resuscitation after dormancy has also been explored, with the elucidation of resuscitation-promoting factors (Lennon and Jones, 2011) and the heat- and desiccation- resilience of seedbanks (Ho et al., 2016a). However, a full understanding of microbial impact at surface-air interfaces demands creative responses to the challenge of studying the inherently slow microbial *activity* in non-saturated environments, in addition to the more popular field of microbial *survival* in non-saturated environments. In a previous study exploring bentonite clay, Stone et al. (2016a) suggested that the combination of high RH and hygroscopic clay surfaces promoted the metabolic activity of microbial communities during desiccation at surface-air interfaces. This continued measurable metabolic activity after desiccation is defined in this study as “metabolic persistence.” In the current study, reminiscent of Brown’s (1976) suggestion that microbes at surface-air interfaces essentially exist in a concentrated solution, the first proposal is that microbial communities at surface-air interfaces can metabolize due to water access (a) from a hygroscopic surface, (b) from the air or (c) from water transfer between the air and the hygroscopic surface. The hypothesis constructed to test this proposal essentially broadens the observation made on bentonite clay (Stone et al., 2016a), and states that if microorganisms can access water interacting with hygroscopic surfaces, then the combination of high relative humidity (RH) and hygroscopic surfaces will increase the metabolic activity of a microbial community at surface-air interfaces, in comparison to low RH and neutral (less hygroscopic) surfaces. This was tested by monitoring microbial community metabolism at desiccated surface-air interfaces, including (a) hygroscopic clay versus sand at both low (30%) and high (75%) RH and (b) hygroscopic polyethylene glycol (PEG) versus plastic at both low and high RH. The microbial community explored constituted desiccation-tolerant organisms, defined in this study as organisms able to survive desiccation either as vegetative cells or via spore-formation. Based on a previous study (Stone et al., 2016a), these species, isolated from a hygroscopic bentonite matrix, were selected for their desiccation tolerance (**Table 1**).

**Table 1:** Strains selected for microcosm inoculation in this study.

Indoor air Isolate^a^	Bentonite bacteria		Bentonite yeasts		Bentonite fungi	
*Arthrobacter* spp. (desiccation- tolerant^a^)	*Bacillus simplex*	*Arthrobacter globiformis*	*Cryptococcus magnus* (extremotolerant^b^)	*Cryptococcus antarcticus* (extremotolerant^c^)	*Penicillium chrysogenum* (Melanized spore-former)	*Fusarium solani* (Spreading hyphae)

*^a^Ronan et al. (2013), ^b^Butinar et al. (2007), ^c^Vishniac and Onofri (2003). One desiccation-tolerant bacterial indoor air isolate was used. All other prokaryotic and eukaryotic strains were selected from bentonite isolates (Stone et al., 2016a).*

The second part of this study explores the idea that higher persistent metabolic rates may render the community more sensitive to the stressors accompanying desiccation. A recent study measured *in situ* microbial growth rates at a single-cell level by labeling membrane fatty acids with heavy water and monitoring the rate of incorporation of fatty acids into cell membranes, demonstrating the vast discrepancy between the typical laboratory and true *in situ* growth rates of *Staphylococcus aureus*, a model opportunistic biofilm species (Kopf, 2015). Similarly, microfluidics has made it volumetrically feasible to explore the slower growth of dental pathogens in sputum, as opposed to the synthetic media that has predominated microbiology for decades (Samarian et al., 2014). These techniques bode a shift in focus to include microbiological studies more representative of natural habitats, despite the slower metabolic rates and the associated experimental challenges. Such ubiquitous natural “slowness” could suggest that there is adaptive strength in microbial metabolic pacing, thus meriting the study of slowly growing organisms in more challenging natural growth conditions. In addition, findings by Stone et al. (2016a) supported previous claims that high RH limits viable cell survival at surface-air interfaces (Ronan et al., 2013), extending this observation to a non-saturated hygroscopic clay matrix. The observations in a clay matrix (Stone et al., 2016a) that (a) microbial metabolic persistence was stimulated short-term at high RH, and (b) viable cell survival was limited long-term at high RH, led to the second hypothesis: that higher initial metabolism correlates with lower long-term resilience, defined throughout this study as a biofilm’s capacity to recover from desiccation, as measured by whole-biofilm CO_2_ generation. To explore this proposed relationship between higher metabolism and lower resilience, the hypothesis states that a microbial biofilm continually pre-exposed to an easily accessible carbon source, with an associated increased metabolic rate, will recover from desiccation less robustly than a biofilm growing under oligotrophic conditions.

The experimental design that was used to test these hypotheses focused on the relationship between microbial metabolic persistence and survival under desiccation, using CO_2_ evolution to track metabolic persistence during desiccation. This involved (1) validating the use of CO_2_ production as a metabolic indicator at surface-air interfaces, (2) searching for further support of the proposition that hygroscopic surfaces and high RH promote microbial communities’ access to water, and (3) demonstrating whether metabolic rates influence the desiccation resilience of a microbial biofilm in both a desiccation-sensitive and a desiccation-tolerant species.

## Materials and Methods

### Desiccation Survival: Relative Humidity, Surface Hygroscopicity, and Pure Culture Cell Viability

Pure culture cell survival at surface-air interfaces of desiccation-tolerant eukaryotes and prokaryotes were assessed using the large droplet method (inoculating with a single droplet rather than a spray), and heterotrophic plate counts according to Jawad et al. (1996) and Ronan et al. (2013). *Arthrobacter* sp., an indoor air prokaryote isolated by Ronan et al. (2013), and *Cryptococcus magnus*, a bentonite eukaryote isolated by Stone et al. (2016a), were grown separately overnight with agitation at room temperature. Bacteria were grown in Tryptic Soy Broth (3 g/L) and yeasts were grown in Yeast Malt Broth (10 g/L dextrose, 5 g/L peptone, 3 g/L malt extract, 3 g/L yeast extract). Media were prepared according to Atlas (2010) and chemicals purchased from Sigma–Aldrich (Oakville, ON, Canada). Both cultures were washed with sterile tap water three times (7,000 g; 5 min) and diluted in sterile tap water to a cell concentration of 10^6^ cells/mL (bacteria) and 10^4^ cells/mL (yeast). Clean glass coverslips were placed in sterile petridishes (6 per dish, 1 mm × 18 mm × 18 mm, VWR International, Mississauga, ON, Canada), inoculated with 50 μL culture per coverslip and allowed to dry in a laminar flow hood for 3 h. For each of *Arthrobacter* and *Cryptococcus*, 75 coverslips were inoculated for incubation at 30% RH, and 75 inoculated for incubation at 75% RH. This procedure was replicated, but the glass coverslips were pre-inoculated with a 50 μL droplet of hygroscopic bentonite clay (100 g/L) solution which was dried in a laminar flow hood for 3 h before inoculation with *Arthrobacter* or *Cryptococcus* and subsequent drying and incubation.

Prior to incubation at low and high RH, the cell viability after drying was assessed in triplicate (*T*_0_). Coverslips, inoculated with *Arthrobacter* or *Cryptococcus* on glass or bentonite, were placed in separate 50 mL Falcon tubes containing 5 mL saline solution (8.9 g NaCl/L), vortexed for 1 min and dilutions plated on Tryptic Soy Agar or Yeast Malt Agar, respectively, for determination of viable cell concentrations per coverslip, as optimized in Ronan et al. (2013). This was repeated at 3 h, 24 h, 48 h and 3, 11, 15, 43, 65, and 234 days. At each time point, triplicate coverslips were assessed for (1) *Arthrobacter* viability at 30% RH and 75% RH, on glass and bentonite, and (2) *Cryptococcus* viability at 30% RH and 75% RH, on glass and bentonite. Viable cell concentrations were represented as a percentage of the cell concentration at *T*_0_ (desiccated inoculum). Means and standard deviations of triplicate coverslips were calculated in Microsoft Excel and plotted in the Veusz plotting package^1^ against time. These programs were used for all statistical analyses and figure plots in this study.

Throughout the study, RH was maintained using saturated MgCl_2_ (33%) and KCl (85%) solutions (100 mL/chamber) in covered 5 L glass chambers, according to Greenspan (1977). RH was monitored with 915 MHz Wireless Temperature Stations (La Crosse Technology, Saint-Laurent, QC, Canada). Bentonite-altered chamber RH, as compared to those reported by Greenspan (1977), stabilized at 23–30% (hereafter referred to as 30%) and 75–79% (hereafter referred to as 75%), consistently returning to the controlled RH within an hour after opening and resealing.

### Desiccation Metabolism: Relative Humidity, Surface Hygroscopicity, and CO_2_ Generation

**FIGURE 1:**
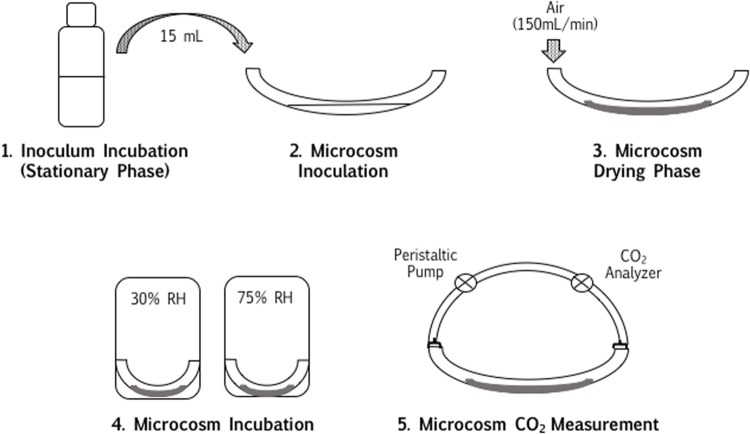
**Closed-loop desiccation metabolic measurements.** Mixed-culture inocula were incubated well into stationary phase, with bentonite, sand, polyethylene glycol (PEG) and tap water, respectively. Inocula were transferred to microcosm tubes [15 mL per tube, in triplicate for low relative humidity (RH) and high RH], and desiccated overnight. Microcosm tubes were transferred to incubators maintained at low (30%) and high (75%) RH. After 3 days of incubation at the respective RH, tubes were connected to a carbon dioxide evolution monitoring system (CEMS) and CO_2_ accumulation was measured in the closed-loop system.

The CO_2_ generation of a mixed culture (**Table 1**; Stone et al., 2016a) desiccated at 30% RH and 75% RH on (1) clay vs. sand and (2) PEG vs. plastic/glass were compared during desiccation. The experiment involved a number of steps, detailed in **Figure 1**. Clay (Fisher, 1923) and PEG-4000 (Dontula et al., 1998) were chosen as hygroscopic substrates, whereas sand (Fisher, 1923) and glass/plastic (Dontula et al., 1998; Harbers et al., 2007) were chosen as neutral substrates.

#### Inocula

Cultures were selected based on a previous study of bentonite clay microbiota (Stone et al., 2016a; **Table 1**). Cultures constituted a mix of desiccation-tolerant organisms, including both vegetative species and spore-formers. Bacteria were grown in TSB and yeasts were grown in YMB, as described above. Cultures were incubated at 25°C with agitation well into stationary phase (2 days, bacteria, 10^7^–10^8^ CFU/mL; 4 days, yeast, 10^4^–10^5^ CFU/mL) and 2.5 mL of each culture combined in four 50 mL conical tubes. Fungal spores were collected and inoculated as described in Stone et al. (2016a), with 2 mL/strain/microcosm (10^2^ spores/mL). Each mixed culture was washed three times with sterile tap water (centrifugation, 7500 *g*, 5 min) prior to microcosm setup. Tap water was selected over physiological saline solution (8.9 g/L NaCl) for a lower precipitated salt concentration upon desiccation. The final mixed culture was combined, diluted 1:3 in tap water, well-mixed and divided between four microcosms in 250 ml Schott bottles, all incubated without agitation at room temperature for 3 days: (1) 100 g/L Wyoming VOLCLAY MX-80 bentonite powder (American Colloid Company, Colony, Wyoming, USA), (2) 100 g/L sand (fine white Ottawa, VWR International, Mississauga, ON, Canada), (3) 100 g/L PEG-4000 Sigma–Aldrich (Oakville, ON, Canada) and (4) pure tap water. Controls included the same four microcosms, suspended in sterile tap water without cells. At day 3, samples from each microcosm were desiccated and incubated at 30 and 75% RH and CO_2_ profiles were assessed for each microcosm, as described below (see CO_2_ Generation). Inocula were incubated into stationary phase, washed and subsequently incubated in nutrient-poor conditions to slow metabolism, representing more natural, starved microbial persistence than typical exponential growth-phase studies.

#### CO_2_ Generation

From each inoculum, 15 mL (well-mixed to include settled matrix in each tube, such as sand or bentonite) was injected separately into 6–10 clean sections of Tygon tubing (30 cm long, 1 cm inner diameter, 15 mL/tube) and non-sterile air passed through at 150 mL/min for 12 h to immobilize the cells on the inside of the tubing. PEG was dried for 18–20 h. Since PEG solubilized entirely during incubation, 1 g was scattered into each microcosm tube after injection of the inoculum as extra matrix media. Bentonite and PEG were evaluated for dryness based on the substrate’s lightness of color and flaking from the surface. Per inoculum, 3–5 tubes were incubated at 30% RH, and three incubated at 75% RH. Airborne contamination was considered irrelevant, since: (1) the cell numbers in the inoculated microcosms were high enough to consider contaminants relatively inconsequential, (2) the study investigated microbial community responses to desiccation and thus pure-culture concerns were not critical, and (3) airborne contaminants are likely to be part of any natural air-surface interface community, and are thus valuable rather than concerning.

After 2 days, CO_2_ production by the desiccated samples was measured by connecting the tubes to a closed-loop carbon dioxide evolution monitoring system (CEMS), described by Kroukamp and Wolfaardt (2009) and Bester et al. (2010), adjusted in this study for desiccated samples as described in Stone et al. (2016a). The carrier gas was ambient air, re-circulated with a peristaltic pump. The accumulation of CO_2_ in the system (3–5 h) was measured after equilibration with ambient air and connection to a sample tube, disconnecting the sample tubes from the CEMS loop and replacing them at controlled RH between measurements.

The evolution of CO_2_ was plotted over time, and the accumulation gradients (ppm CO_2_/min) were compared to un-inoculated controls of each respective matrix, to account for variable matrix CO_2_ absorption capacities (Stone et al., 2016b). Means and standard deviations of gradients of 3–5 replicate samples were calculated in Excel and compared with a Mann–Whitney *U* test for non-parametric data.

#### CO_2_ Generation: Controls

To verify that CO_2_ gradients were due to microbial metabolism, a number of controls were included:

(1) One set of inoculated bentonite tubes, incubated after desiccation for 7 days at 30% RH and 75% RH, respectively, were connected to the closed-loop CEMS system directly after re-wetting with 3 mL sterile tap water per tube,
(2) One set of inoculated PEG tubes, incubated after desiccation for 7 days at 30% RH and 75% RH, respectively, were connected to the closed-loop CEMS system directly after wetting with 3 mL sterile tap water per tube, and
(3) A set of bentonite tubes, one inoculated and one sterile control, incubated after desiccation at 75% RH, were connected to the closed-loop CEMS system directly after rewetting with 3 mL sterile TSB (3 g/L) per tube.

The gradients were compared to those recorded during desiccation, with the expectation that water would increase the CO_2_ accumulation gradient in inoculated tubes, but not in control tubes, and TSB would increase the gradient even more markedly.

### Desiccation Metabolism and Resilience

**FIGURE 2:**
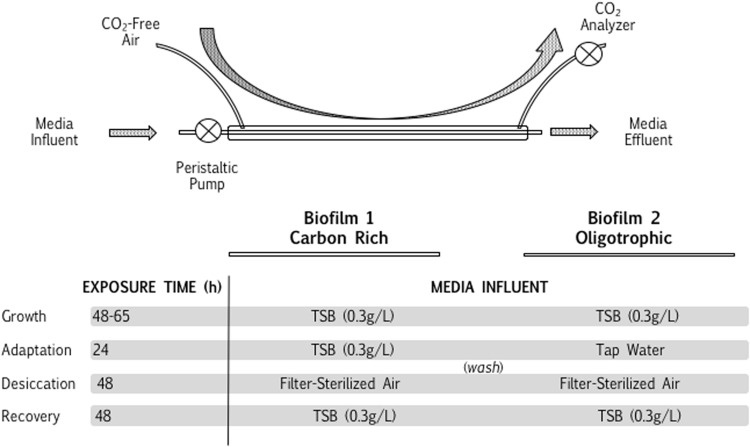
**Open-loop desiccation metabolic measurements.** The influence of oligotrophy on the desiccation responses of either (a) *Arthrobacter* or (b) *P. aeruginosa* PAO1 were assessed using an open-loop CEMS system. Two identical biofilms were grown to a metabolic steady state, and subsequently exposed to distinct nutrient sources (carbon-rich = TSB; oligotrophic = tap water) prior to washing and subsequent desiccation. The metabolic rate during recovery of each biofilm was measured using a single-pass CO_2_ system, as opposed to the accumulation in **Figure 1**, and the metabolic rate during recovery was normalized against the initial biofilm metabolic rate for comparisons between biofilms.

The influence of biofilm metabolic rates on biofilm desiccation resilience (capacity to recover) was studied in biofilm flow systems, in both a desiccation-tolerant and desiccation-sensitive bacterial species. *Pseudomonas aeruginosa* PAO1 (desiccation-sensitive; Ronan et al., 2013) and *Arthrobacter* (desiccation-tolerant; Ronan et al., 2013) biofilms were grown in continuous systems and open-loop carbon dioxide generation was monitored as an indicator of biofilm activity. A schematic visualization of the experimental setup is detailed in **Figure 2**. Continuous flow systems were assembled according to Bester et al. (2010). Briefly, two biofilm flow systems were sterilized with ethanol (1 h) followed by a diluted (2 in 10) commercial bleach solution (3 h). The system was washed (37.5 mL/h) with sterile tap water overnight (10 h), equilibrated with 0.3 g/L TSB in tap water (1 h) and each system was inoculated identically with either *Arthrobacter* or *Pseudomonas* cells upon stopping the flow of media (0.5 mL, ~10^6^ cells/mL, grown in TSB for 14 h). After a 45 min (*Pseudomonas*) or 6 h (*Arthrobacter*) attachment period, medium flow was restarted. At 50 h (well into the stationary phase of the CO_2_ profile), the medium of one biofilm was changed to sterile tap water. The two identical biofilms were continuously exposed to (a) tap water (oligotrophic) or (b) 0.3 g/L TSB in tap water (carbon-rich), respectively, for 24 h and subsequently run dry. Before running dry, 3 mL of sterile tap water was run through both biofilms at a higher rate (2.5 mL/min) to wash the TSB biofilm. Both biofilms had filter-sterilized air delivered (5 L/min) for 2 days. The desiccated biofilms were then re-exposed to TSB (0.3 g/L), with a 30 min incubation period, and the recovery respiration rate, measured with CO_2_ production and normalized as a percentage of the pre-media change biofilm respiration rate, was used as an indicator of post-desiccation biofilm resilience. The CO_2_ molar flow rate during biofilm desiccation was also compared between oligotrophic and carbon-rich conditions. The experiment was repeated in triplicate, for each pair of *Pseudomonas* and *Arthrobacter* biofilms. The (a) desiccation rate during desiccation and (b) resilience (post-desiccation respiration rate as a percentage of initial biofilm respiration rate) were compared between oligotrophic and carbon-rich biofilms per species, using a Student’s *t*-test of independent means for normally distributed data, with a confidence interval of 95%.

## Results and Discussion

### Desiccation Viability: Relative Humidity and Surface Hygroscopicity

**FIGURE 3:**
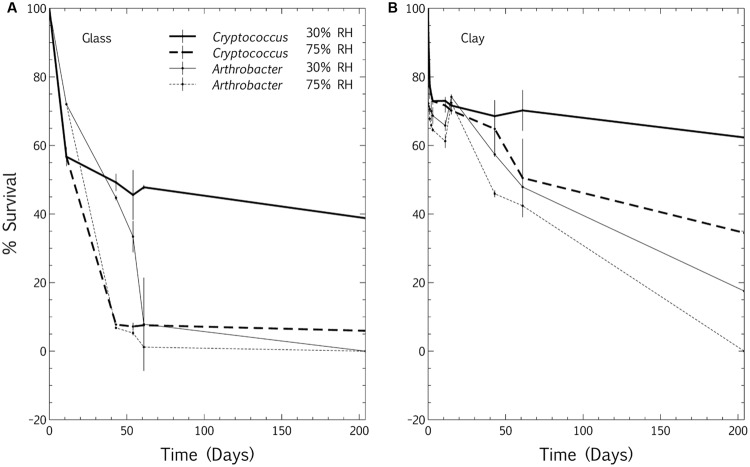
**Relative humidity, hygroscopicity and the survival of pure-culture prokaryotes and eukaryotes at surface-air interfaces.** The survival of cells inoculated onto **(A)** glass coverslips and **(B)** glass coverslips pre-inoculated with a coat of desiccated bentonite was evaluated over a period of months. Survival was expressed as a percentage of the desiccated inoculum evaluated after a 3 h drying period. A desiccation-tolerant yeast (*Cryptococcus magnus*) was compared to a desiccation-tolerant prokaryote (*Arthrobacter* sp.), and survival was contrasted at low (30%) RH and high (75%) RH. Plots represent means of triplicate samples and error bars the standard deviation.

Following inoculation with the large-droplet technique, the survival of both the eukaryote and prokaryote was extended at bentonite-air interfaces in comparison to glass-air interfaces, at both high and low RH (**Figure 3**). As bentonite provides more carbon and trace elements than clean glass (Karnland et al., 2006), such an extension of survival is expected. Also, the eukaryotic isolate, *C. magnus*, demonstrated better viability under desiccation than the prokaryote, which was an indoor air isolate selected specifically for its reported desiccation-resistance (Ronan et al., 2013). The extended viability of *Cryptococcus* at both bentonite- and glass-air interfaces, as compared to *Arthrobacter*, confirms several reports of increased desiccation tolerance in eukaryotes (**Figure 3**; Brown, 1976; Morano, 2014). However, the trend of viability inhibition at high RH remained consistent throughout this study, despite the nature of both the surface and the inoculum. Thus, the conclusions of Ronan et al. (2013) were confirmed and extended to include a desiccation-tolerant eukaryote and hygroscopic clay surfaces. This also supported the long-term survival trends of natural prokaryotic and eukaryotic populations in dry bentonite reported in Stone et al. (2016a).

Microbial viability at surface-air interfaces is extended at low RH and suppressed at high RH, despite species variation (Turner and Salmonsen, 1973; Walters et al., 2005; Ronan et al., 2013). Although clays are a well-known buffer against environmental stressors, a recent study of bentonite clay microbiota showed that the suppression of microbial viability at high RH occurs within such a clay hygroscopic matrix too, suggesting that surface-bound water does not decrease the impact of RH on microbial viability. Using heterotrophic plate counts to assess the natural microbiota of bentonite after a year of dry incubation at low (30%) and high (75%) RH, both eukaryotic and prokaryotic populations were consistently significantly less viable at high (75%) RH than at low (30%) RH (Stone et al., 2016a). Ronan et al. (2013) also showed, using the large-droplet method, that the survival of a desiccation-tolerant indoor air isolate (*Arthrobacter* sp.) was significantly inhibited at high (75%) RH. This technique was employed in the current study to extend the above-mentioned observations, exploring the combined influence of surface hygroscopicity and water vapor on microbial longevity at surface-air interfaces. Due to its application as a sealing material in deep geological repositories for nuclear waste (Stroes-Gascoyne et al., 2011), as well as the broader environmental occurrence of clays in soil and wetland environments, Wyoming MX-80 bentonite montmorillonite clay was chosen as the primary hygroscopic microbial substrate in this work. In order to extend the work of Ronan et al. (2013) to include eukaryotes and hygroscopic surfaces, the conclusions were extended to include the same desiccation-tolerant *Arthrobacter* sp. air isolate, as well as a desiccation-tolerant eukaryotic bentonite isolate (*C. magnus*) on both glass coverslips and on glass coverslips coated with hygroscopic bentonite clay.

### Desiccation Metabolism: Relative Humidity and Surface Hygroscopicity

**FIGURE 4:**
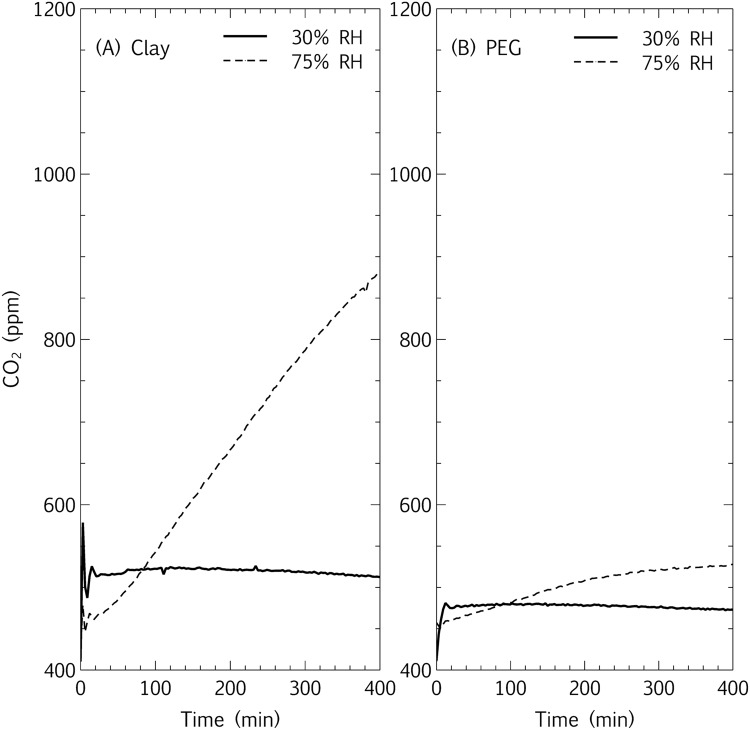
**Interface microbial metabolism: relative humidity and hygroscopic matrices – single microcosms.** Carbon dioxide production at **(A)** bentonite-air interfaces and **(B)** PEG-air interfaces, measured in a closed-loop CEMS system. Samples were inoculated with microbial biomass (mixed community, **Table 1**) that had been pre-incubated under static conditions for 2 days on the respective substrate (bentonite and PEG), dried overnight, and incubated at low (30%) RH and high (75% RH) for 48 h before connecting to the CEMS system for measurement of CO_2_ accumulation.

**FIGURE 5:**
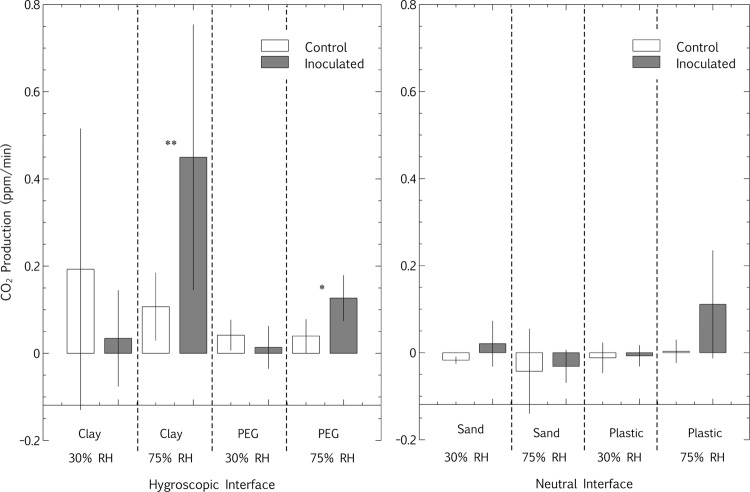
**Interface microbial metabolism: relative humidity and hygroscopic matrices – averages.** Enriched microbial biomass (mixed community, **Table 1**) was inoculated in suspension with the two hygroscopic substrates (Clay and PEG) and two neutral substrates (Sand and Plastic/Glass), along with sterile controls. The inocula were statically incubated for 2 days, transferred to tubes, desiccated overnight and incubated at low (30%) RH and high (75%) RH for 2 days. The rate (gradient of single microcosms such as **Figure 4**) of carbon dioxide accumulation was compared between samples. Bars represent means of 3–5 replicate samples, and error bars represent the standard deviation. The difference between the means of inoculated and sterile controls were statistically calculated with a Mann–Whitney *U* test, and significant differences are indicated (*p < 0.05, **p < 0.005, **Table 2**).

Spore survival and viability is often explored in desiccated conditions, but even vegetative bacteria can survive for years, challenging our understanding of bacterial physiology and metabolism (Otter et al., 2015). In addition, the metabolic state of viable but non-culturable organisms has yet to be elucidated. On this note, many of the organisms explored in this study – including the desiccation tolerant species – are vegetative non-spore formers, including *Arthrobacter*, *Cryptococcus*, and *Pseudomonas*, highlighting the emphasis of this study on metabolic persistence in relation to long-term viability. In contrast to survival inhibition in bentonite at high RH, the above-mentioned study (Stone et al., 2016a) also showed that the hygroscopic clay-air interface allowed for increased short-term CO_2_ production of a microbial community at high (75%) RH. However, microbial metabolic CO_2_ production was below the closed-loop CEMS detection limit at low (30%) RH, even at the hygroscopic clay interface. This observation led to the proposal that hygroscopic surfaces improve the access of microbial cells to water vapor at surface-air interfaces. The hypothesis designed to test this states that if a hygroscopic matrix improves microbial access to water vapor at surface-air interfaces, then both clay and PEG-4,000 at high (75%) RH will stimulate higher CO_2_ production in microbial communities at surface-air interfaces than less hygroscopic surfaces such as sand and plastic. Of the eight combinations of neutral (sand and plastic) and hygroscopic (clay and PEG) surfaces inoculated with a mixed microbial community (**Table 1**) and incubated at high and low RH, the accumulation of CO_2_ in a closed-loop CEMS (**Figure 1**) was only observable for microbial communities at clay-air interfaces at high (75%) RH and PEG-air interfaces at high (75%) RH (**Figures 4** and **5**; **Table 2**). For all un-inoculated controls, the production of CO_2_ was approximately zero and all inoculated microcosm tubes incubated at 30% RH – irrespective of surface hygroscopicity – were not significantly higher than the controls, according to a Mann–Whitney *U* test for non-parametric data (**Figure 5**; **Table 2**). Any slightly negative CO_2_ production rates are likely due to the matrix CO_2_ absorption, which is related to pH and RH at surface-air interfaces (Stone et al., 2016b). On both sand and plastic surfaces, there was no significant difference between the means of CO_2_ production (ppm/min) between samples incubated at 30% RH and 75% RH. Microbial communities desiccated on a bentonite surface and incubated at high (75%) RH exhibited a mean CO_2_ production rate of 0.45 ppm/min, significantly higher than a sterile control sample (**Figure 5**; **Table 2**). The mean CO_2_ production rate of the same microbial community on PEG was lower than bentonite (0.13 ppm/min), but still significantly higher than the mean sterile PEG control (**Figure 5**; **Table 2**).

**Table 2:** Relative humidity and microbial respiration at surface-air interfaces.

Control/inoculated paired microcosm matrix	*U*	*p*
Clay 30%	12	0.458
Clay 75%	8	0.008*
PEG 30%	1	0.174
PEG 75%	2	0.014*
Sand 30%	1	0.063
Sand 75%	3	0.256
Plastic 30%	4	0.414
Plastic 75%	2	0.138

Differences between inoculated matrices and un-inoculated controls were assessed with a Mann–Whitney *U* test of independent means for non-parametric data. The *u* values (*U*) and probability values (*p*) are compared, with significance indicated* at a confidence interval of 95%.

The hypothesis that the combination of hygroscopic surfaces and high RH extended microbial metabolism at surface-air interfaces was thus supported, with significantly microbial CO_2_ production occurring only at high RH on bentonite and PEG (**Figures 4** and **5**; **Table 2**). In both cases, only the combination of hygroscopic interfaces and high RH significantly promoted microbial metabolism during desiccation (*p* < 0.05).

The prolonged metabolism at bentonite-air interfaces as compared to PEG-air interfaces is again likely due to the mineralogical properties of bentonite, providing the microbes low levels of carbon and trace elements (**Figures 4** and **5**; Karnland et al., 2006). In addition, the microbial community comprises predominantly isolates of bentonite origin (Stone et al., 2016a), likely more adapted to bentonite than PEG. The porosity and increased surface area of bentonite and PEG, as compared to sand and plastic, are intimately related to the hygroscopic capacity of these matrices. It has been shown that laboratory mineral dissolution rates are generally exaggerated due to higher clay-surface reactive sites than natural clay deposits (Maher et al., 2006). Similarly, the activity of truly dry soil microbial populations is often difficult to extricate from the activity of microbial populations in occluded water within the soil (Stotzky and Pramer, 1972). A more fundamental link between hygroscopic surfaces, water vapor and microbial activity could be further pursued on flat surfaces coated with smooth chemical hygroscopic compounds, however, these ‘interfering’ variables of porosity and surface area are characteristic of many hygroscopic surface-air interfaces in nature and are thus more relevant to this study. Although this work did address the challenge of measuring slower growth and metabolic rates at surface-air interfaces, as opposed to the better-studied rates in liquid culture, there are elements that were not representative of *in situ* scenarios. High cell concentrations was the primary unnatural parameter in most of the surface-air interface analyses, likely more representative of biofilms present in the richer environments of nosocomial, wastewater or food industries – many of which are exposed to periods of desiccation – rather than organisms that land on surfaces during air or vector dispersion.

#### Measuring Desiccation Metabolism: Controls

**FIGURE 6:**
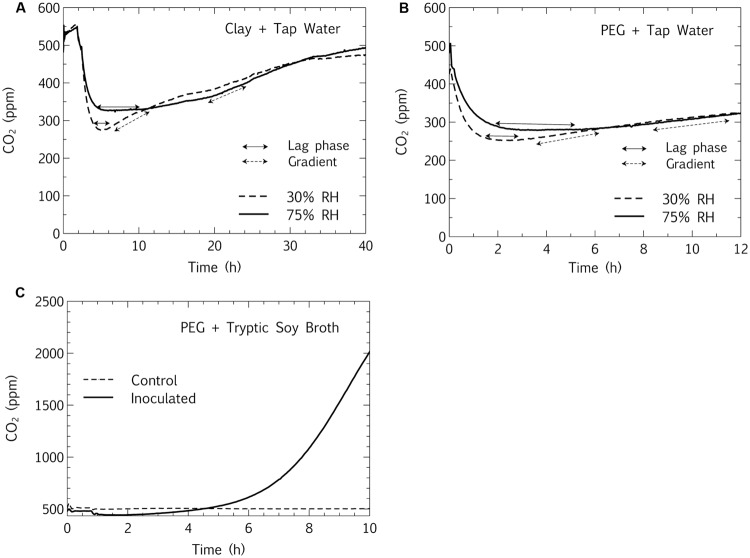
**Metabolic CO_2_ profiles upon rewetting of desiccated samples.** Desiccated microbial communities at bentonite **(A)** and PEG **(B)** interfaces were incubated at low and high RH for 7 days, rewet (*T*_0_) with tap water and CO_2_ accumulation was measured in a closed-loop system. An inoculated PEG sample was compared to a sterile PEG sample by rewetting (*T*_0_) with a rich carbon source **(C)**. Ambient CO_2_ levels (450–550 ppm) are evident at *T*_0_ (moment of connection to the closed loop system upon rewetting after 7 days of desiccation), with no CO_2_ gradient in the tubes prior to rewetting (data not shown).

Since the conclusions of persistent metabolism at hygroscopic surfaces and high RH are based on CO_2_ production measured by a relatively recent technique, controls were designed to demonstrate that the CO_2_ accumulation gradients observed in the closed-loop CEMS were, in fact, due to microbial metabolism and not purely chemical reactions. The hypothesis was that if the CO_2_ produced within the closed-loop system was representative of microbial metabolism during desiccation, then: (1) the addition of water would increase the CO_2_ accumulation gradient at both 30% RH and 75% RH, (2) the addition of a rich carbon source (3 g/L TSB) would increase the gradient even more markedly, and (3) sterile controls would show no increase in metabolism upon rewetting with water or added nutrient. The addition of tap water to inoculated samples incubated at 30 and 75% RH at clay and PEG interfaces increased the gradient (**Figures 6A,B**). Prior to rewetting, the gradients (CO_2_ accumulation) of all samples, which had been desiccated for 7 days at the respective relative humidities, had dropped to zero (data not shown). After rewetting, the CO_2_ profiles fluctuated from ambient levels (450–550 ppm). An initial negative CO_2_ gradient was due to the highly basic (pH 9–10) bentonite slurry acting as a CO_2_ sink (Stone et al., 2016b). The CO_2_ solubilization was markedly greater in microcosms incubated at 30% RH than at 75% RH, likely due to some pre-solubilization of CO_2_ in clay-bound water in hygroscopic matrices incubated at high RH. Similarly, the addition of TSB to microbial communities desiccated at both bentonite (data not shown) and PEG interfaces led to an even more dramatic increase in CO_2_ production rate than the addition of tap water, with zero CO_2_ production in sterile controls (**Figure 6C**). Thus, the positive slopes represent renewed metabolic activity in desiccated microcosms, with the maximum activity dependent on nutrient availability. Notably, in hygroscopic matrices the lag phase was approximately twice as long (4 h vs. 2 h, respectively, **Figure 6A**; and 2.5 h vs. 1 h, respectively, **Figure 6B**) for samples incubated at high (75%) RH than at low (30%) RH, metabolically corroborating the survival data that shows lower viability at higher RH (**Figure 3**). This suggests that the viability inhibition at high RH is linked both to cell death as well as dormancy mechanisms, since the difference in lag phases is only 2 h. It is unlikely that cell growth alone is responsible for the short time interval necessary for high-RH microcosms to reach a similar metabolic steady state as the low-RH microcosms after rewetting. Elucidating viable and total (microscopic) cell numbers in relation to these metabolic profiles would be a logical extension to this study. Similar *in situ* spikes in CO_2_ production upon rewetting of dried agricultural and wild soils have been extensively documented, and the phenomenon has been coined the “Birch Effect” (Jarvis et al., 2007). The results from the present study suggest that the RH at soil-air interfaces may influence the Birch Effect, with the slower metabolic recovery at high RH, potentially due to the inhibition of microbial viability at high RH (**Figures 3** and **6**).

### Desiccation Metabolism and Survival: Biofilm Resilience

**FIGURE 7:**
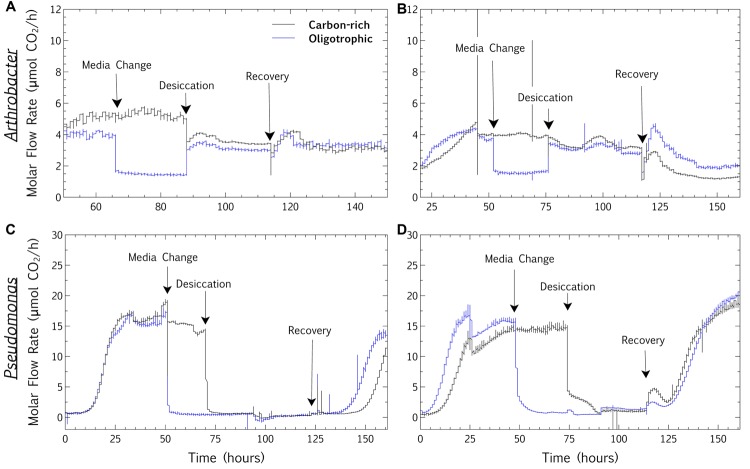
**Carbon and whole-biofilm post-desiccation: metabolic profiles.** Individual metabolic profiles demonstrated observable trends in response to desiccation in *Arthrobacter* (**A,B**, replicates of desiccation-tolerant biofilms), but not in *Pseudomonas aeruginosa* (**C,D**, replicates of desiccation-sensitive biofilms). Both biofilms were grown on TSB (0.3 g/L), but whilst one biofilm was grown continuously on this media (carbon-rich), the other was changed to tap water (oligotrophic) at “Media Change” (for approximately 24 h). The molar flow rate of CO_2_ production was higher for carbon-rich, metabolically active *Arthrobacter* biofilms during desiccation, than for oligotrophic biofilms **(A,B)**. In contrast, the resilience (recovery respiration rate, normalized for individual biofilms over the pre-desiccation respiration rate) was higher for oligotrophic *Arthrobacter* biofilms. The molar flow rate of CO_2_ production for *Pseudomonas* showed no observable trends or differences in desiccation respiration or resilience **(C,D)**.

**FIGURE 8:**
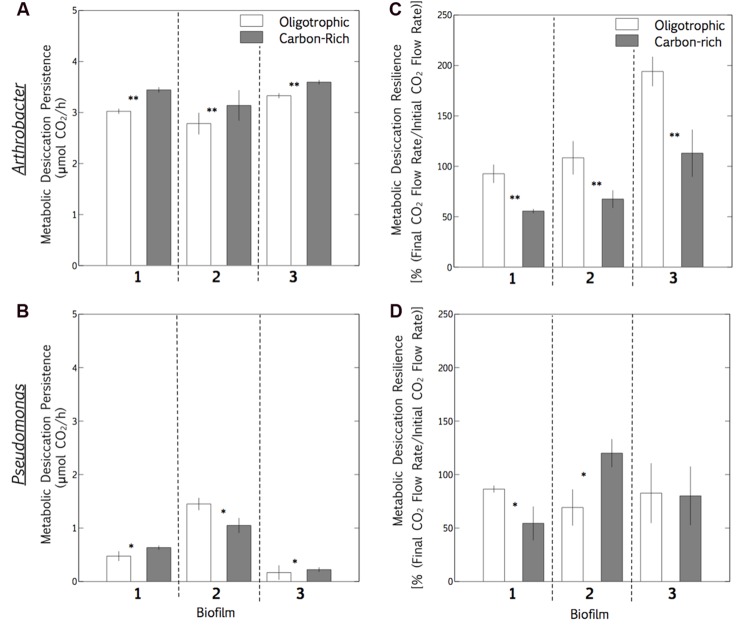
**Carbon and whole-biofilm post-desiccation resilience: metabolic averages during desiccation and recovery.** The means of three individual experimental systems (**Figure 7**, two biofilms per experiment, six biofilms per species) were compared for *Arthrobacter* and *Pseudomonas aeruginosa*, in order to quantify observable trends in their respiration response to desiccation. The mean respiration rate (5–10 h) of carbon-rich and oligotrophic biofilms was compared **(A,C)** during the desiccation period, and **(B,D)** during the post-desiccation recovery period (normalized as a percentage of the pre-desiccation whole-biofilm respiration rate). Observable trends were clear in *Arthrobacter* biofilms, but not in *Pseudomona*s biofilms. Columns represent means of a 5–10 h period for a single biofilm (during or post-desiccation), and bars represent standard deviation. The difference between carbon-rich and oligotrophic biofilms were calculated with a Student’s *t*-test of independent means, and consistent significance (*p* < 0.05) trends are indicated**, as well as significant (*p* < 0.05) differences without a consistent trend* (**Table 3**).

The final hypothesis expounded on the observations that microbial survival is inhibited at high RH, whereas short-term metabolic persistence is improved at high RH. The hypothesis, linking these survival and metabolism observations, proposes that if a higher persistent metabolic rate during desiccation lowers the survival of a desiccated microbial community at surface-air interfaces, then an oligotrophic biofilm will demonstrate a higher metabolic desiccation-recovery capacity (termed resilience in this study) than a metabolically active biofilm. This hypothesis was evaluated in both a desiccation-tolerant (*Arthrobacter*) and desiccation-sensitive (*P. aeruginosa)* species. In *Arthrobacter* and *P. aeruginosa* biofilms, initial growth for 2 days, starvation for 1 day and desiccation for 2 days allowed for a full recovery of both starved and metabolically active biofilms, facilitating a comparison of respiration profiles during desiccation and recovery with online CEMS (**Figures 2**, **7**, and **8**). In each experiment, both biofilms were grown on TSB well into a steady-state metabolic profile. Subsequently, one biofilm was continuously fed TSB (carbon-rich), whilst the other was changed to tap water (oligotrophic) at the “Media Change” point, for approximately 24 h (**Figure 7**). After washing and desiccation, the resilience (metabolic recovery from desiccation, normalized against the pre-desiccation biofilm metabolic rate) was compared between the carbon-rich and oligotrophic biofilms. **Figure 7** demonstrates the CO_2_ profiles of two individual replicate biofilms per experiment, whereas **Figure 8** is a summary of the means of three replicates per experiment. For desiccation-tolerant *Arthrobacter* biofilms, CO_2_ generation persisted during desiccation of a carbon-rich biofilm, whereas the oligotrophic biofilms showed greater resilience (recovery respiration rates, normalized for each individual biofilm as a percentage of the pre-desiccation respiration rates), supporting the hypothesis (**Figures 7A,B**). The average respiration rate during desiccation was consistently significantly lower for an oligotrophic biofilm in independent triplicate scenarios (**Figure 8A**; **Table 3**), whereas the average respiration rate post-desiccation (normalized against the pre-desiccation respiration rate) was consistently significantly higher for oligotrophic biofilms in independent triplicate systems (**Figure 8B**; **Table 3**). Significant differences, with a confidence interval of 95%, were calculated per biofilm with a Student’s *t*-test of independent means (**Table 3**). In contrast, the desiccation respiration rates and post-desiccation respiration rates showed no consistent trends in triplicate *P. aeruginosa* biofilms (**Figures 7C,D** and **8C,D**; **Table 3**), suggesting that within these experimental parameters, metabolic activity prior to desiccation has no influence on the resilience of a desiccated *P. aeruginosa* biofilm.

**Table 3:** Differences between oligotrophic and carbon-rich biofilms, in terms of mean desiccation respiration rate and mean post-desiccation resilience.

	Respiration during desiccation			Normalized respiration recovery/resilience		
Carbon/oligotrophic paired biofilm	*t*-value	df	*p*-value	*t*-value	df	*p*-value
*Arthrobacter* 1	49.621	198	<0.00001	-21.066	198	<0.00001
*Arthrobacter* 2	53.374	198	<0.00001	-28.772	198	<0.00001
*Arthrobacter* 3	11.750	198	<0.00001	-36.386	198	<0.00001
*Pseudomonas* 1	16.112	198	<0.00001	-13.165	198	<0.00001
*Pseudomonas* 2	-24.573	198	<0.00001	16.308	198	<0.00001
*Pseudomonas* 3	4.514	198	<0.011	-1.316	198	0.19171

*The t-value and degrees of freedom (df) were generated for the normally distributed data sets with a Student’s *t*-test of independent means, at a confidence interval of 95%.*

These distinctive respiration profile responses in desiccation-tolerant versus desiccation-sensitive species indicate that the hypothesis might play a role in biofilm adaptation to desiccation, in addition to morphological and physiological adaptations. The higher CO_2_ production rate of *Pseudomonas* than of *Arthrobacter* (both pre- and post-desiccation) when grown on TSB supports studies showing that the species-specific utilization of the carbon source has a distinct effect on both metabolic rate and adaptive resilience (Jackson et al., 2015). As previously suggested, “the altered morphology and/or growth patterns of bacteria growing at low humidity might be more ecologically relevant than their textbook appearance at high humidity since their natural habitats are often dry” (Lidwell and Lowbury, 1950). Longer time periods of starvation may facilitate physiological adaptation in relation to metabolic rates, and microscopy could narrow this window by direct observation of such morphological adaptation. Another valuable extension of the study would be to assess the response of mixed community biofilms to the same starvation and desiccation conditions. In a study bringing the osmolyte accumulation hypothesis into question *in situ* in soils (Kakumanu et al., 2013), adaptation to desiccation was more clearly linked to population diversity and dynamics than osmolyte accumulation or cell turnover, suggesting the importance of species studied. Furthermore, desiccation cycles and gradients (de Goffau et al., 2009) are representative of natural environments and could be incorporated into similar studies, since the impact of cycles such as the circadian rhythm on metabolism is extensive (Kovac et al., 2009; Trinder et al., 2015; Ho et al., 2016b).

Previous studies of microbial populations in dust showed that lower temperature decreased the thermodynamic potential for high metabolic rates, thus lowering the aging of a population and extending survival rates (Lidwell and Lowbury, 1950). Similarly, high (80–90%) RH stimulates microbial metabolism in house dust, as well as the growth of spore-bearing fungi (Korpi et al., 1997). Also, RH approaching saturation levels demonstrated some short-term increase in viable cells in dust microbial populations, prior to the typically reported long-term inhibition of viability at high RH (Lidwell and Lowbury, 1950). These authors proposed that the mechanism of inhibition at high RH was the concentration of salt ions dissolved at the hygroscopic interface. However, it is possible that metabolic persistence at high RH could mirror the negative thermodynamic influence of metabolically favorable temperatures on cell viability. Persistent metabolism could prevent desiccation-adaptive mechanisms, or higher metabolic rates during the initial stages of desiccation could decrease the total long-term nutrients and energy available to the community.

There is conflicting evidence regarding the influence of metabolic rates on the desiccation tolerance of biofilms. Some studies have shown that higher carbon fluxes lead to thicker biofilms, better suited to adaptation and stress responses (Condell et al., 2012). Others have rather emphasized adaptation due to stress exposure, claiming that the extensive morphological and physiological responses to desiccation are “not just indirect responses to stress, but rather responses of these cells to become more stress resistant” (de Goffau et al., 2009). Some of these morphological SOS responses include down-regulation of autolysin (leading to a lower growth rate) and the accompanying filamentation and decrease in cell separation, and increases in cell size, cell wall thickness and antibiotic resistance. At high RH more cell division is evidenced (de Goffau et al., 2009), supporting the proposal that persistent metabolic activity is linked to increased water availability and lower long-term viability.

Slow growth rates within biofilms have also been linked to increased antibiotic resistance (Walters et al., 2003; Otter et al., 2015). Slow growth may be sustained in biofilms by cell death (programmed or natural), predation or cannibalism. The concept of bacterial cell suicide was initially viewed with skepticism, since bacteria were considered single-cell organisms. However, increased evidence of programmed cell death within biofilms has strengthened the concept of a biofilm as a “whole organism” (Bayles, 2014). This type of terminology has also been used within medicine, referring to the gut microbiome as an “organ” (Trinder et al., 2015). Thus, viewing a biofilm as a whole organism capable of metabolic pacing increases the legitimacy of the theory that cell death and nutrient cycling could contribute to survival and metabolism at surface-air interfaces. In this scenario, an environment that stimulates persistent faster metabolism would deplete accessible nutrients faster. Similarly, predation has been shown to influence metabolic regulation in biofilms, with ciliate predation of *Cryptococcus* biofilms preventing stagnation and stimulating persistent metabolism (Joubert et al., 2006). Thus, slow metabolism based on cell death could be a direct adaptive response linked to community desiccation resilience. Since this SOS response of programmed cell death might be directly linked to growth rate and viability (Bayles, 2014), an interesting future study would be to compare the rates of programmed cell death and predation at high and low RH. Bester et al. (2010) demonstrated that in a saturated carbon-fed system, planktonic cell output for proliferation only costs the biofilm 1.0 ± 0.2% of the total carbon turnover. Within a liquid system, these cells contribute to biofilm spawning. However, within a desiccated biofilm – reflecting a closed nutrient system – such cell production could maintain a low level of persistent metabolism due to natural cell lysis, predation, cannibalism, or cell suicide. Regulation of the rate of this metabolism would thus be an effective adaptation to desiccation. The observation of Ronan et al. (2013) that cell viability during desiccation is greatly improved by the large droplet method as opposed to the spray method supports this contention of nutrient cycling within a population.

Diverse experimental parameters pose a challenge in the mining of hospital, food preparation and built ecology literature for support or contradiction of the results presented here. However, the survival of methicillin-resistant *S. aureus* after desiccation was lower at 40% RH than at 16% RH, across varying surface properties (glass, wood, plastic, cloth; Coughenour et al., 2011). Similarly, Worth Calfee and Wendling (2012) demonstrated that low temperature and RH enhanced the survival of *Brucella suis* on various building materials. These studies support the premise of this work, that higher RH might lead to increased microbial access to water vapor, promoting faster metabolic activity and inhibiting long-term survival by decreasing the total energy available to the community. High RH also improved the transfer efficiency of five bacterial, phage and viral species, on both porous and non-porous surfaces (Lopez et al., 2013). The authors concluded that since transfer efficiency is better under wet than dry conditions, the morphological activity necessary to promote transfer might be sustained at higher RH due to microbial access to water, also supporting the proposal of the current study that high RH promotes persistent microbial metabolism at surface-air interfaces. Similarly, airborne dispersion has been significantly and positively correlated to RH (Obbard and Fang, 2003). Again, assuming that there is an energy cost to microbial dispersion (morphological shifts that are energy expensive), this also supports the findings of this study. The antimicrobial activity of hygienic paint, containing silver nanoparticles and titanium dioxide, against *Escherichia coli* and methicillin-resistant *S. aureus* was greater at high RH than at low RH (Dominguez-Wong et al., 2014). Since low metabolic rates are correlated with higher antibiotic resistance, the hypotheses are further supported.

## Conclusion

High (75%) RH was shown to inhibit long-term (200 days) cell viability in prokaryotic and eukaryotic populations on both neutral and hygroscopic clay surfaces. A combination of high RH and hygroscopic surfaces promoted short-term (<4 days) measurable respiration of microbial communities at surface-air interfaces, whereas microbial metabolic persistence was not evident at neutral surfaces at high RH, or at any surface-air interfaces at low RH. The addition of water and carbon to the desiccated communities confirmed the CEMS closed-loop CO_2_ accumulation gradient to be representative of microbial metabolism. Finally, the proposal that whole-biofilm desiccation resilience may be improved by oligotrophy was only supported in a desiccation-tolerant *Arthrobacter* species, and not in a desiccation-sensitive *Pseudomonas* species.

The interactive influence of RH and surface properties on the metabolic persistence and long-term viability of microbial populations was demonstrated. Both of these properties have application in natural biogeochemical cycles, nuclear waste management, nosocomial infections and industrial settings, with (1) increased viability promoting the potential of a community to influence its surroundings upon rewetting, and (2) persistent, slow metabolism facilitating the potential for long-term corrosion, dissolution, fermentation, the deposition of metabolites or biogeochemical cycling in natural environments during desiccation. Particularly, the influence of oligotrophy, or metabolic rates, on biofilm or community resilience might inform the design of many laboratory-based biofilm adaptation studies. An appreciation of the microbial ability to harness their matrix for access to water vapor, and the relationship between metabolic persistence and long-term survival, is applicable in the monitoring and engineering of industrial environments, and is an intriguing picture of the response of these versatile organisms to the vast challenges within which they so remarkably persist.

## Author Contributions

WS executed all experimental work, wrote the manuscript and was involved in the inspiration and design of the hypotheses and experiments. OK provided in-depth guidance in the inspiration, design, and execution of the research. OK edited the manuscript from an engineering and microbiology perspective. JM provided guidance from a geoscience perspective and edited the manuscript. DK and GW facilitated the collaborative project between the NWMO and the universities, and provided guidance and editing in the fields of microbiology and biochemistry.

## Conflict of Interest Statement

The authors declare that the research was conducted in the absence of any commercial or financial relationships that could be construed as a potential conflict of interest.

## Footnotes

1 http://home.gna.org/veusz/
